# User Acceptability and Perceived Impact of a Mobile Interactive Education and Support Group Intervention to Improve Postnatal Health Care in Northern India: A Qualitative Study

**DOI:** 10.21203/rs.3.rs-3320095/v1

**Published:** 2023-09-18

**Authors:** Valentina Cox, Preetika Sharma, Garima Singh Verma, Navneet Gill, Nadia G Diamond-Smith, Mona Duggal, Vijay Kumar, Rashmi Bagga, Jasmeet Kaur, Pushpendra Singh, Alison M El Ayadi

**Affiliations:** University of California, San Francisco; Postgraduate Institute of Medical Education & Research; Postgraduate Institute of Medical Education & Research; Postgraduate Institute of Medical Education & Research; University of California, San Francisco; Postgraduate Institute of Medical Education & Research; Survival of Women and Children Foundation; Postgraduate Institute of Medical Education & Research; Indraprastha Institute of Information Technology Delhi; Indraprastha Institute of Information Technology Delhi; University of California, San Francisco

**Keywords:** India, antenatal, group care, mHealth, mobile phone, postnatal, postpartum

## Abstract

**Background:**

Postnatal care, crucial for preventing and assessing complications after birth, remains low in India. An interactive mHealth community-based postnatal intervention was implemented to promote healthy maternal behaviors through knowledge and social support in rural Northern India. However, there is limited information on how virtual health interventions in resource-constrained settings are perceived by the users and which elements influence their engagement and sustained participation.

**Objective:**

We explored the user perceptions of acceptability and impact of a virtual interactive maternal and child health intervention pilot tested in Punjab State, India, including their perspectives on barriers and facilitators to engage with this intervention.

**Methods:**

This qualitative study was embedded within extensive mixed-method research, and oriented by the Realist Evaluation approach. Sixteen participants were recruited from the parent study. They were identified by purposive sampling to cover diverse levels of attendance and engagement with the intervention. In-depth interviews were conducted by phone. Following translation, a framework analysis was completed to search for the main themes. Feedback was requested from intervention moderators during the process to prioritize local interpretation.

**Results:**

Study participants reported overall satisfaction with the intervention. The mothers appreciated the educational material provided and the communication with other participants and health professionals. Across context, intervention, and actor domains, the barriers most commented on were network and connectivity challenges, lack of time due to household responsibilities, and feeling uncomfortable sharing personal experiences. Family buy-in and support were fundamental for overcoming the high domestic workload and baby care. Another facilitator mentioned was moderators’ guidance on using the different intervention modalities. Regarding perceived impact, participants shared that MeSSSSage increased their capability and motivation to breastfeed, seek care as needed, and use contraception according to their preferences. Finally, participants suggested adding more topics to the educational content and adjusting the dynamics within the group calls to improve the intervention.

**Conclusions:**

This study identifies the high acceptability and perceived impact of a novel postnatal care program in a rural setting, including the users’ perceived barriers to engaging with the intervention and possible solutions to overcome them. These findings enable refinement of the ongoing intervention, providing a more robust framing for its scalability and long-term sustainability. On a larger scale, conclusions from this research provide new insights and encouragement to global stakeholders who aspire to improve maternal and neonatal outcomes in low-income and middle-income countries through mHealth.

## Introduction

### Maternal and newborn health in India

India has been recognized for its exceptional progress in maternal and infant health. It has reduced maternal mortality ratio by 70% in less than 25 years, from 556 per 100,000 live births in 1997–8 to 97 in 2020 [1,2]. Similarly, the country has decreased the under-five mortality by 65%, from 111 per 1,000 live births in 1990 to 39 in 2018 [3].

Despite this success, much more is needed to achieve the Sustainable Development Goals (SDGs) for maternal and child health in India [4]. Access and utilization of postnatal care, crucial for preventing and assessing complications after birth, remains low [5]. Approximately one-fifth of mothers and newborns did not receive any postnatal services within 24 and 48 hours of delivery in the country between 2019 and 2020 [6,7]. Inadequate access is explained by several reasons: low levels of mobility in married women, insufficient linguistic abilities from health professionals, and cultural barriers, among others [8,9].

The use of mobile technology for health (mHealth) has shown an ability to surmount many of the barriers mentioned above [10]. With increasing availability of mobile phones and access to the internet, mHealth has been used widely to improve maternal and infant health, for example, by enhancing skilled attendance at birth, immunization, and facility-service utilization, including postnatal care [10–15].

Simultaneously, community-based interventions have proven to be a low-cost initiative to advance maternal and neonatal outcomes in South Asia [16]. The community-based models aim to improve health service provision by considering the cultural and social environment of the patients [17]. For maternal health, evidence has been gathered about the benefits of using community as a resource, for example, through support groups [17–20]. Recent studies have shown that peer groups can be particularly beneficial for women during pregnancy and postpartum, offering the opportunity to share their emotions and concerns in a safe space, allowing them to normalize and validate their experiences while learning from one another [16,19,21].

In India, mHealth initiatives have been implemented in various geographical areas targeting varied health care outcomes [22,23]. Research has shown that mobile phone-based job aids for health workers, and text messages as reminders for maternal visits, have increased the number of maternal health care check-ups, and improved newborn nutrition, and immunization in hard-to-reach settings [22,23]. In addition, programs based on supportive women’s groups guided by health care professionals demonstrated a decrease in neonatal mortality in an underserved rural area in eastern India [24]. Despite these advances, little has been studied about using both strategies in postnatal care, mHealth with a community-based approach, to improve maternal and newborn health care outcomes in this country.

#### MeSSSSage: a novel intervention for rural India

In November 2020, a mobile interactive education and support group intervention: *Maa Shishu Swasthya Sahayak Samooh* (MeSSSSage) or “Maternal and Child Health Support Group”, was developed in rural Punjab, Northern India. In this region, maternal and infant mortality remain at high levels: 167 per 100,000 live births and 49 per 1,000 live births, respectively [25]. In contrast, current average maternal mortality and infant mortality are 145 and 35 per 100,000 live births in the country [7]. In addition, only 82% of mothers and 79% of infants receive postpartum care, roughly half of the infants are breastfed exclusively for six months, and 49% of women use a modern family planning method [25].

The MeSSSSage intervention aims to improve infant and maternal health by promoting certain healthy maternal behaviors such as breastfeeding, immunization, and appropriate care-seeking during the postpartum period. It provides culturally appropriate maternal and child health information through a mobile app or an interactive voice recognition system (IVR) from the last trimester until six months after birth. In addition, it facilitates communication with health providers and other mothers through 20-minute to one-hour-long virtual weekly meetings and a group text chat (WhatsApp^®^), both moderated by community health workers. One physician (obstetrician or pediatrician) participates in one call each month for each group.

**Figure 1:**
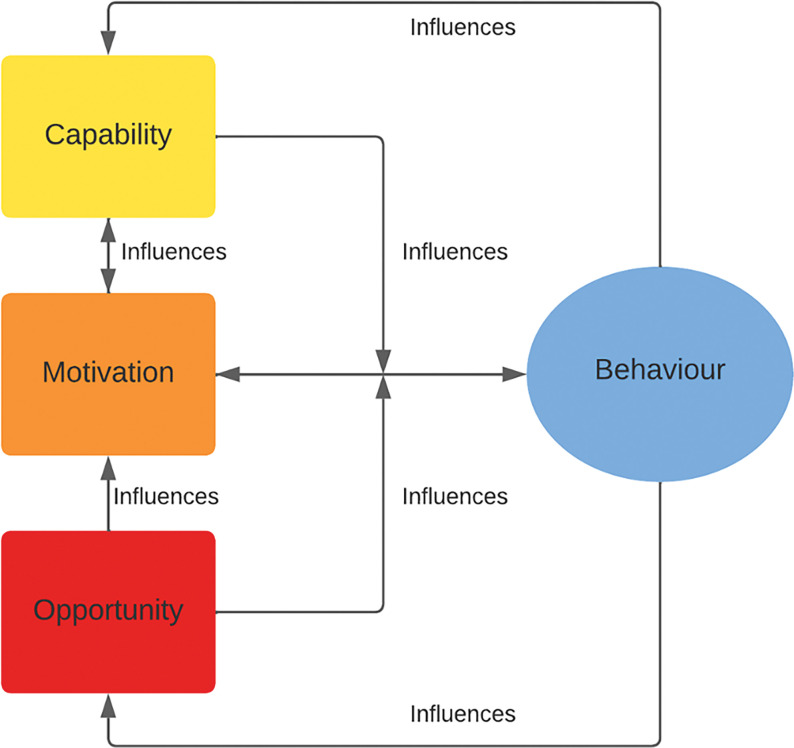
The COM-B Model theoretical framework.

The intervention is rooted in the COM-B model theoretical framework (Figure 1). This model proposes that three components influence behavior (B). First, capability (C), the person should have the necessary knowledge and psychological and physical skills [26]. Second, opportunity (O) the context provides an adequate and accessible environment for executing the behavior [26]. And third, motivation (M), the new behavior is attractive and aligned with personal goals [26]. MeSSSSage intends to work through these components by strengthening knowledge and social support and providing the opportunity to communicate directly with health professionals.

Figure 1. A graphical depiction of the interaction between the components that influence Behavior: Capability, Motivation, and Opportunity, by the COM-B framework. Adapted from West and Michie, 2020 [27].

MeSSSSage was developed iteratively from formative research on women’s health education and social support needs in the perinatal period, in combination with Indian and WHO perinatal care guidelines. Subsequently, a 6-week preliminary pilot test was performed to optimize intervention content and modality [28]. The larger intervention was piloted from September 2021 through July 2022 among eight groups of 20 women, with three different combinations of modalities (mobile app or IVR, with or without virtual meetings, and the group text chat) [28].

#### Fulfilling the gaps

Although mHealth and social support interventions have been studied in several contexts, gaps in the literature remain regarding how these programs are perceived by the users and which elements influence user engagement and sustained participation, particularly in low literacy settings like rural Punjab [10, 24, 29].

Previous research has shown that mechanisms that influence mHealth engagement among pregnant women are satisfaction and usefulness or perceived impact of the intervention [30, 31]. These topics are considered subdomains of user acceptability [30, 32]. Acceptability is crucial for health programs to penetrate better into the population and be sustainable in time [31]. For MeSSSSage, acceptability is also acknowledged by users’ participation and the barriers and facilitators implicated in the process [28].

In the context of the current MeSSSSage pilot phase, exploring user acceptability will provide relevant information about how participants interact with the different components of the program. At a local level, this knowledge will help to refine MeSSSSage and the implementation process. At a global level, the evidence collected may provide new insights for understanding the factors involved in users’ participation, helping to design better mHealth interventions for women and newborn postpartum care in rural areas.

## Methods

### MeSSSSage Evaluation Study Design

A qualitative method, specifically in-depth interviews, was chosen as the ideal approach to explore the acceptability and impact of the virtual interactive maternal and child health intervention MeSSSSage and appropriately capture the complexity and nuances of user perceptions [30, 33, 34].

**Figure 2:**
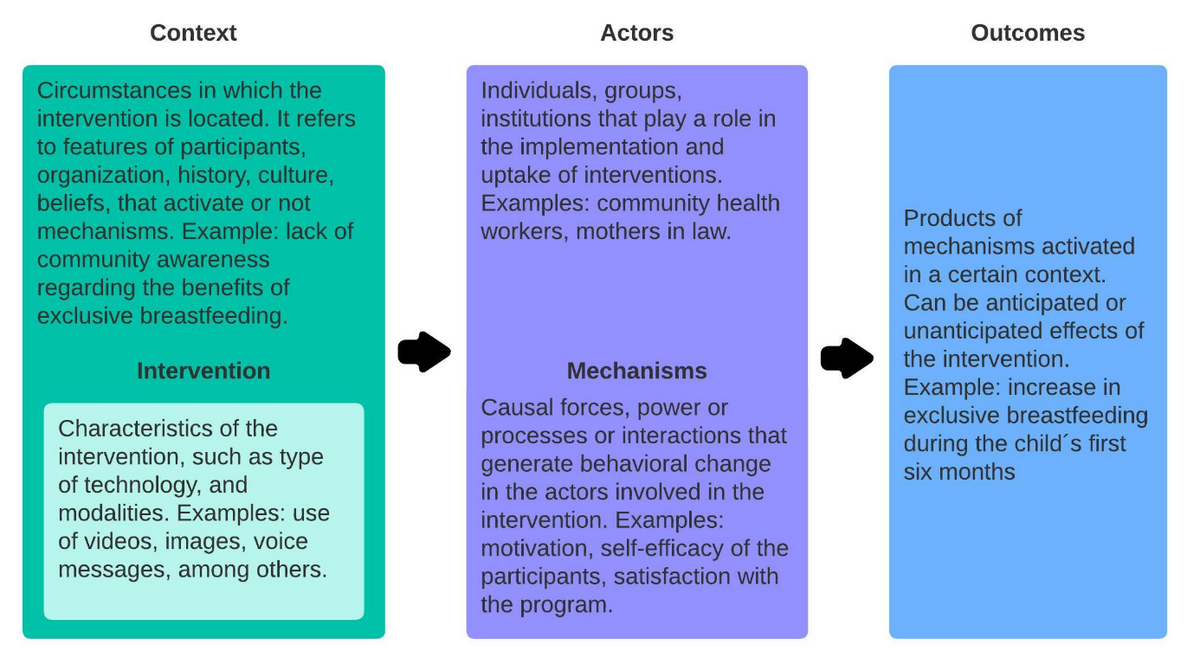
Realist framework diagram.

This qualitative study was embedded within a larger mixed-methods research study analyzing feasibility, acceptability, and preliminary intervention effectiveness. The qualitative study is oriented by a Realist Evaluation approach. First described in 1997 by Pawson and Tilley, Realist Evaluation seeks to understand what, why, and how interventions succeed or fail, which is fundamental for the refinement process of MeSSSSage [35]. Later adapted by other implementation science experts, this model recognizes interventions as “theories put into practice” by actors in a specific *context* that can trigger *mechanisms* toward certain *outcomes*, as depicted in Fig. 2 [35].

Previous research in maternal and newborn health has shown the usefulness of the Realist Evaluation to generate a better understanding of the processes involved in changing stakeholder behavior while acknowledging the influence of contextual factors [34]. In this study, this framework provided a comprehensive view of the elements influencing user acceptability, from the context, the actors, and the intervention itself, thereby recognizing possible mechanisms that affect the achievement of promoting healthy maternal behaviors targeted.

Figure 2. Brief explanation of the elements involved in the Realist Evaluation framework: context, mechanisms, and outcomes. Own elaboration from data obtained from Westhorp G et al, 2011; and Kabongo et al, 2021 [30, 33].

### Study Participants

Sixteen study participants were identified from the parent study by using a purposive sampling technique to achieve a variety of perspectives from the different groups and modalities of MeSSSSage [36]. Members of the research team (GS, NG) recruited two users from each group by phone calls, covering diverse levels of attendance and engagement with the intervention. Feasibility constraints, such as the number of interviewers available for this study and study timeline, also defined the number of participants. Inclusion criteria from the parent study considered women who were pregnant at time of recruitment, of any parity and any gestation number, that spoke the local language (Punjabi or Hindi), and over the age of 18. Women were excluded from the parent study if they did not have a personal mobile phone or were unwilling to accept one from the study team, given participant engagement in the intervention occurs over mobile phone.

### Data Collection

An in-depth interview guide (Appendix 2) was developed through an iterative process by the research team (PS, MD, GS, NG, ND, AE, VC). Topics of the interview considered the above mentioned aims of the study: overall satisfaction with the intervention and the MeSSSSage components, emphasizing social support and the educational content, and barriers and facilitators for engaging in the group calls and the chat groups. Additionally, questions regarding the usability of the mobile application were constructed by using previous validated survey questionnaires found in literature [37–39]. Finally, questions regarding the perceived impact of MeSSSSage were added, probing the different modalities of the intervention. To align with local cultural norms, Indian members of the research team (PS, MD, GS, NG) reviewed the questions of the in-depth interview guide before the data collection.

During the recruitment process, women who met the study inclusion criteria were contacted telephonically by the study researchers and verbally confirmed their informed consent for study participation. Appointments were scheduled according to participant availability. Semi-structured interviews were conducted by members of the research team (GS, NG) by phone, eliminating possible participation barriers such as lack of transportation or difficulties leaving home [40]. The interviews were audio-recorded with participant permission and lasted between 20 to 45 minutes. The interviews were conducted in Punjabi or Hindi and transcribed and translated to English for analysis.

### Data Analysis

Data analysis employed an inductive approach, framework analysis and was performed iteratively.

Framework analysis is a systematic process used in qualitative methodology that involves a pre-defined number of stages according to Ritchie and Spencer: familiarization, identifying a thematic framework, indexing, charting, and interpretation [41, 42]. This approach emphasizes thematic conclusions rather than theoretical development and is therefore particularly useful for identifying and explaining associations between experiences and attitudes. Framework analysis has been used previously in women’s health research to evaluate user experiences with maternal and infant health mobile applications [43, 44].

In this qualitative evaluation of MeSSSSage acceptability, the Realist Evaluation model informed the process of generating deductive codes for satisfaction, and barriers and facilitators for engaging the intervention. For perceived impact, codes were created considering the framework in which MeSSSSage is rooted, the COM-B model. Subsequently, familiarization with the data was performed by reading the transcripts multiple times. Then, new inductive codes were added iteratively during the analysis as new topics of interest were generated. Afterward, indexing and charting were performed to find main themes and subthemes using Dedoose and Microsoft Excel, respectively.

This analysis represents the work of a cross-cultural and multi-disciplinary team including a Chilean physician (VC), American public health researchers (ND and AE), Indian physicians (MD, VK, RB), Indian public health researchers (PSh, GS, and NG), and Indian information technology researchers (JK and PSi). To ensure that local interpretation was prioritized within the diverse cultural backgrounds of the context and research team members, video conference meetings were organized to establish the codebook (PS, MD, GS, NG, AE, ND, VC), Then, presentations to the rest of the research team of the parent study (VK, RB, JK, PSi) were performed monthly to verify the themes and subthemes found and discuss the interpretation of findings. Finally, a report was written (VC) and shared with the rest of the research team for peer review and final feedback.

## Results

### Participants Characteristics

Sixteen women were interviewed in the study, accounting for 10% of the total users of MeSSSSage intervention. Participants were between 21 and 35 years old, married, and the majority lived in an intergenerational household, over half of them with their mother-in-law. There was a narrow range of educational attainment, with all participants having completed at least primary education (5^th^ grade). Four participants reported living in a household classified as below the poverty line. For detailed sociodemographic data, see Table 1.

Women were interviewed between four and six months postpartum, half of them participated in the intervention during their first pregnancy. Regarding phone access, all participants had their own personal phone. However, two-fifths shared their phone daily with someone else. Before MeSSSSage, almost all women used the phone for text chat (WhatsApp), half of the participants used it for voice calls, and six of them to watch videos.

### Exploring Acceptability: User Satisfaction with MeSSSSage

Overall, MeSSSSage was valuable to the mothers interviewed. The participants were satisfied with the educational material delivered. In addition, sharing with other participants, the intervention moderators and health care providers, was perceived as useful to their mothering. Further details, thematic analysis and quotations are presented in Appendix (Multimedia Appendix 3).

#### Satisfaction with the educational component

In general, participants were satisfied with the educational component of MeSSSSage. Quantity and quality of the information provided, as well as language spoken on the mobile app and the group calls, was acceptable for all the participants. In addition, the opportunity to talk directly to doctors was highly valued by the participants who engaged in the group calls.

“Mam, I already have one baby. At that time, I used to think, “I wish I had someone to share my doubts with”. I like the things in the group. There is a doctor team, proper guidance, and participants are the same as us. If we have some problem or confusion, we can contact you. Before we couldn’t go outside, we asked our query to our mother, grandmother, and then did according to their advice.”

(Participant 12, Group call + IVR + WhatsApp)

“Earlier (before MeSSSSage) we weren’t able to clear some queries from doctors because they don’t have much time and we also don’t have much time, but in the group or in the app, we are able to clear our doubts and whenever we feel, then we start play the recording, that is how our doubts get cleared, it was so helpful. […] I am satisfied, like I get a call from PGI (MeSSSSage moderators), then I ask my questions and clear them out. I am waiting with excitement for the 3 pm call.”

(Participant 14, Group call + App + WhatsApp)

### Satisfaction with the group calls

Overall, the participants involved in the group calls were satisfied with the social support component of the intervention. They appreciated the safe space to share their concerns and motherhood experiences with other women and the intervention moderators, resolving their queries and validating their feelings and thoughts. In addition, the figure of doctors participating in some of the group calls was highly valued by all the participants and their families.

“Mam, we have suddenly become mothers. Mothers have questions and, in those questions, we also come to know about our baby, as some problems are similar. From the answers they receive we come to know what should be done.”

(Participant 1, Group call + App + WhatsApp)

“They (group calls, IVR messages) normalize all the things, that it’s ok. Initially, we said one, two months is ok [to rest] then you get normal. Then I thought I have not been well for one and a half months, what should I do now? Then you said it’s ok for six months, take time to heal yourself and body changes, take some rest and normalize the things, then I feel relaxed. I felt panic because of difficulties, fear, and a backache, but after this program, I feel comfortable.”

(Participant 12, Group call + IVR + WhatsApp)

“Experience was good because we wait for a call to ask question and it was amazing, I never heard that doctors call the patients and I experience that, doctors call us and without needing to go anywhere questions are resolved on calls.”

(Participant 14, Group call + App + WhatsApp)

### Acceptability: Barriers and Facilitators to engagement with MeSSSSage

Across context, intervention, and actor domains, the barriers most commented on were network and connectivity challenges, lack of time due to household responsibilities, and feeling uncomfortable to share personal experiences or ask questions. Family buy-in and support were fundamental facilitators for overcoming high domestic workload and baby care, and the intervention moderator’s guidance for the download and use process of the different technology modalities. A summary of these findings is presented in Table 2.

### Barriers from the context: Connectivity and household responsibilities

Limited network connectivity was the main barrier for almost half of the participants, affecting various activities, such as downloading the app properly, listening to IVR calls, and participating in the discussion during the group calls. For example, one participant mentioned not being able to receive any feedback on her questions during a group call, which resulted in her ultimately leaving the meetings altogether.

“Sometimes I am unable to hear the voice over video call. I was diabetic at that time, and I put queries but neither got any response nor proper voice quality, so after that I didn’t join the meeting.”

(Participant 15, Group call + App + WhatsApp)

In addition, eleven participants mentioned having difficulties joining the group calls or listening to the content delivered by IVR calls or the app because of their high workload in the household and domestic responsibilities. These challenges were highlighted by those with children already. For group calls, childcare was the primary reason for not attending, posing different challenges to engagement with MeSSSSage between the antenatal and postpartum periods.

“I had attended [the group calls] during pregnancy only. After delivery, I didn’t attend. I have two children. Now the elder one is four years old and the second one is little. With childcare and household work, I didn’t get any time for this meeting.”

(Participant 15, Group call + App + WhatsApp)

“After my child [was born] I did not get time and we are farmers and have a lot of work in the fields. […] We are two sisters-in-law and we both delivered children with a gap of almost two months, so we have no one to take care of the babies. Our mother-in-law is no more, so one takes care of the children and the other takes care of the household, so we barely get time to see our phones.”

(Participant 11, App)

### Barriers from the intervention: Technical issues

Regarding barriers coming from the intervention, half of the participants in the app modality groups mentioned having problems downloading the app, regardless of the effort of the intervention moderators to help with the process through multiple calls if it was necessary. One user was unable to download the app at all. Other mothers overcame difficulties by asking for more assistance from the intervention moderators and family members.

“Mam, it took some time (to download the app), and I also got a call from you, saying that I should use another number and then download it.”

(Participant 5, App)

### Barriers from the intervention: Promoting interaction between mothers

The social support component of the MeSSSSage intervention relies heavily on the interaction between group members. For participants joining the group calls, the structure of the calls, including the presence of other mothers, was mentioned before the intervention started. However, it was not clear to one participant that she could communicate and contact other users beyond the intervention spaces, even when she was already participating in the group call meetings and the chat group with the assistance of her husband.

“I - Did you ever talk to another participant?

R - No mam, I have spoken only to the doctors.

I - How do you think we can improve your relationship with the other participants?

R – Mam, we can directly talk to [ the other women] on call sometimes. I don’t have any idea about the [WhatsApp] group, as my husband did, but can we have the contacts of other participants?”

(Participant 6, Group call + IVR + WhatsApp)

### Barriers from the actors: User literacy and perception of comfort

One of the obstacles participants noted to joining the intervention was lower literacy. For example, one participant preferred videos and audio material because she couldn’t understand the written messages.

In addition, varying levels of comfort during the group calls was a factor limiting women’s engagement with the intervention. For example, one participant perceived competition between the group members. She also mentioned a lack of privacy because the husband of one of the users participated in the group call, even when this was highly discouraged by the intervention moderators. Both the sense of competitiveness and the presence of a male reduced her engagement. Other women expressed being shy and therefore felt apprehensive about sharing personal experiences or asking questions in front of other users. Where participants were uncomfortable posing specific questions to the group overall, they described reaching out privately to the intervention moderator to get resolution.

“Involvement of each [group member] is low. We didn’t talk like friends; we are just comparing ourselves with each other, like she has a beautiful baby, so I will not share a picture of my baby. Because of these thoughts, mothers are not involved in the group […]. Her husband attended the call because she was not present if you remember [….] He shared very useful information. I appreciate the father’s involvement, but being a female on call or WhatsApp, it felt it breaches privacy. It was very uncomfortable for me to share personal things in the presence of a male.”

(Participant 12, Group call + IVR + WhatsApp)

“I sent the query about sex and relationship [privately to the intervention moderator]. I know they are questions that all may have, but I didn’t feel comfortable to ask in front of everyone.”

(Participant 13, Group call + IVR + WhatsApp)

### Facilitators from the context: Family buy-in

Family member acceptance and willingness to actively support women in the intervention were fundamental in encouraging the participants to stay engaged in the different modalities of MeSSSSage. In addition, the idea of being able to ask questions from the comfort of home, especially during the Covid-19 pandemic, was highly valued by families. Husbands and mothers-in-law were the most mentioned figures, helping with technical assistance and household responsibilities.

“[My husband] helped me to download and join the group, and said it is good for you, keep active on the group.”

(Participant 9, Group call + App + WhatsApp)

“He [my husband] was very supportive and would say that I should leave the chores and attend the call. No matter you are late by one hour, just attend and listen to what the doctors are teaching for you and our child.”

(Participant 10, Group Call + App + WhatsApp)

“[My mother-in-law] supports me a lot because she knows that instead of taking the baby outside to consult the doctor, it is better to discuss everything over the call and post it in the group. These things she likes the most, that we get instantly a solution.”

(Participant 13, Group call + App + WhatsApp)

“[My family] encourages me [to participate], because due to Covid we are not able to visit the hospital and the clinic, and this makes it better for us; we get all solutions over the call.”

(Participant 14, Group call + App + WhatsApp)

“It [MeSSSSage] was very much welcomed by my family. They said that we get the information directly from doctors, so they are happy about it.”

(Participant 8, Group call + IVR + WhatsApp)

### Facilitators from the intervention: Flexibility to deliver information

WhatsApp, the mobile app platform for the group chats used during the MeSSSSage intervention, presents different features, such as sharing videos and audio messages that can be seen or heard as many times as desired. Most women highly valued receiving material that could be reviewed at any hour.

“Mam, we don’t have time to answer the [IVR] call. [We are] unable to listen properly during household work. When you send videos instead of calls, it is good according to me […] those videos are nice, and I think it’s beneficial for me. For the call we need to make time, but we can play those videos in our free time.”

(Participant 13, Group call + IVR + WhatsApp)

### Facilitators from the actors: Intervention moderator’s assistance and user motivation

The intervention moderator’s guidance was fundamental to improving participation and correct use of the different modalities involved in the MeSSSSage intervention.

“So, [the moderator] explained to me about the Zoom app and how to attend the call. She explained everything to me very properly.”

(Participant 1, Group call + App + WhatsApp)

The willingness of the women to engage in the different modalities of intervention, explained by the high level of motivation to increase their knowledge about their newborns and themselves, facilitated their engagement with MeSSSSage.

“Mam, I did not have very high expectations from the program, but as soon as it went on, I really learned a lot and had lots of information. I used to wait for Tuesday for the call.”

(Participant 7, Group call + IVR + WhatsApp)

“Every time when we get the call, the [intervention moderators] provide information regarding [mother] and baby. We like this, and problems get solved from your advice. In the app we get all the information on how to check baby movements, it motivates us.”

(Participant 14, Group call + App + WhatsApp)

### Perceived impact of MeSSSSage: increased knowledge and skills for care

During the interviews, most of the participants reflected on how MeSSSSage changed their perceptions regarding motherhood, increasing their capability and motivation in various topics, particularly taking care of the baby, breastfeeding, seeking care for the baby, and family planning. These effects were seen even in women who already had children before the intervention.

“Because of the group, I am able to take care of the baby in a better way […] yes mam, like they say we can apply ghee on the naval area of the child, but you on the calls mentioned that one should not apply ghee.”

(Participant 3, Group call + IVR + WhatsApp, first child)

“I learned many things, that breast milk is sufficient for the child, and it is a complete food, and artificial milk should be avoided.”

(Participant 8, Group call + IVR + WhatsApp, first child)

“You always guide us to take a gap [before becoming pregnant with the] next child or you should use some contraceptive. It’s fine; we are very much conscious about it and get motivation from your side.”

(Participant 12, Group call + IVR + WhatsApp, second child)

The participants also mentioned how they shared what they have learned with their family members and other community members.

“In the family someone had a baby, then I used to tell the baby’s mother these things we have learned from the program. On baby rashes she used cream over it, then I told her to use coconut oil [instead].”

(Participant 13, Group call + IVR + WhatsApp)

“In our neighborhood there was a young baby, so I shared information about her medicines with her mother.”

(Participant 14, Group call + App + WhatsApp)

### User recommendations for MeSSSSage

Various ideas on how to improve MeSSSSage and its different modalities were obtained throughout the interviews. Most of the suggestions referred to adding more topics to the educational content, and a few related to adjusting dynamics within the group calls.

In terms of timing of the intervention, one participant expressed that MeSSSSage should start earlier during pregnancy to increase its impact.

Regarding the different modalities, the participants using IVR, meaning that they received calls to receive information instead of using the mobile app with audio messages, asked for more flexibility in delivering information using other apps or resources. This idea was also encouraged by users with both IVR and WhatsApp chat groups.

“Mam do not send [the IVR call] again and again, but on WhatsApp you can send the information. We can listen to it according to our preference when we have time.”

(Participant 4, Group call + IVR + WhatsApp)

In addition, some suggestions for increasing participation during the group calls and managing the question period within the call were shared by two participants. One interviewee suggested that the question period within the group call be more structured, with intervention moderators adding more educational content in the absence of specific questions:

“When you ask us if we have any questions, at that time you should randomly choose any topic related to child and mother and tell us about that. Sometimes things are not discussed and hence skipped.”

(Participant 10, Group Call + App + WhatsApp)

The other interviewee that made recommendations promoted the use of diverse activities such as games to increase interaction between the users:

“You can do little activities like games for around five to ten minutes, or song suggestions, damsharas (guess the movie name). It [will increase the] involvement of group members. Or share your handiwork/crafts, like when you are free with your baby and knitting sweater, cooking, gardening, and we appreciate them and feel like friends.”

(Participant 12, Group call + IVR + WhatsApp)

Finally, almost half of the participants had recommendations on topics for the educational content of the ongoing intervention, relevant to both maternal and child health. For maternal health, suggestions tended to be related to the postpartum period, asking for more information about postpartum bleeding, mental health, diet, and sexuality after childbirth. Two participants wanted to add material during pregnancy addressing miscarriages.

“[Spontaneous] abortion has not been told about, and after, nothing was told about the [sexual life] after the delivery. Something new should be added in it which we be able to understand because we don’t know anything about the problems that a woman face.”

(Participant 13, Group call + IVR + WhatsApp)

For newborn care, mothers asked for guidance on monthly growth, skin care, and developmental milestones.

“We did not know what the weight and height of the baby should be according to the months. We do not know anything. The baby suddenly gains or loses weight, and we hardly have any knowledge about it.”

(Participant 3, IVR)

“I think the care of the skin of the child should be a topic of discussion as the skin of my child is changing and I should have the information.”

(Participant 6, Group call + IVR + WhatsApp)

## Discussion

### Principal Results

This qualitative analysis explored the user acceptability and perceived impact of MeSSSSage. This novel mHealth intervention aims to increase engagement in health-promoting behaviors such as breastfeeding practices, postpartum contraceptive initiation, and appropriate care-seeking for emergent maternal or infant illness by providing tailored knowledge and social support to women during the postpartum period. The findings from our qualitative inquiry offer user-based insights to review and refine the intervention and highlight important considerations for decision-makers interested in implementing this type of health program in similar settings.

Overall, MeSSSSage participants reflected high satisfaction with the intervention, especially the usefulness of the educational content. For those who participated in the group calls, the presence of doctors was highly valued by both the users and their families.

In addition, interacting with the intervention moderators, and other women through the different modalities of MeSSSSage increased participants’ capability and motivation to adopt behaviors important for optimizing maternal and child health, as well as gave them the confidence to help other mothers facing similar challenges within their community.

Despite broad satisfaction, participants also recognized difficulties in engaging with MeSSSSage, across the context, the intervention itself, and the actors involved, all of which can be categorized within technological and socio-cultural factors. Moreover, it is acknowledged that some of these are beyond the margins of action of the health interventions. Nevertheless, recognizing the barriers from the user’s perspective and suggestions could improve access to the target population and identify which individuals might require additional resources and attention.

Regarding technological challenges, one main barrier from the context was connectivity. This reality is particularly relevant in India, where access to network remains limited to roughly 40% of the country despite rapid proliferation of mobile device ownership, with nine of ten phone users facing poor wireless connectivity [45]. However, previous literature from developing countries has found that partnering with local authorities and non-governmental institutions might overcome this situation and secure sustainable digital infrastructure and capacity for mHealth to succeed [46]. Along those lines, considerations should be made regarding network accessibility of potential participants and understanding the possible limitations of implementing MeSSSSage in the most remote locations. The scalability of this program is anticipated to be improved by Government of India’s Digital India Programme which seeks to promote greater network access across the country, particularly in rural geographies [47].

The challenges participants faced in downloading the MeSSSSage application are consistent with technical difficulties experienced by other application-based interventions in resource-constrained settings [48]. For MeSSSSage and other mHealth interventions, considering the technological skills and experience of the target audience and preparing intervention moderators to address that possible gaps will be critical for its long-term sustainability and eventual scalability in other areas of India [49].

Sociocultural factors including social norms around household responsibilities and childcare represented another challenge to intervention engagement for participating mothers. Patriarchal gender norms that validate a male-breadwinner female-caregiver model are standard in India as in other low and middle-income countries [50]. Encouraging flexible access to information through chat group platforms like WhatsApp, which are widely used among the Indian population, including those who use feature phones, might respond better to these cultural constraints [51, 52]. Though IVR has been used in many previous interventions in India and elsewhere, newer technologies, like WhatApp, were preferable to respondents and should be prioritized in the future. MeSSSSage could increase the use of this modality even more in the future to deliver educational content, ensuring that in terms of access, the participants have smartphones or feature phones that are adequate to this type of mobile applications.

Our findings regarding family member interest in peripheral engagement in the MeSSSSage intervention support the broader acceptability of this intervention and guide potential avenues for increasing users’ participation and expanding intervention reach. Previous research confirms that direct engagement of household decision-makers or social support providers may increase intervention impact, demonstrating positive effects on postpartum maternal and neonatal health when husbands and mothers-in-law participate in educational interventions [53–55]. For example, in rural Nepal, a short intervention for couples of two antenatal educational sessions of 35 minutes resulted in a significant increase in postnatal checkups compared to women who received the sessions alone [50, 55]. For the MeSSSSage intervention, this could be done by promoting exclusive group calls in which male partners could join. In this way, the participation of husbands could be promoted while protecting the privacy and comfort of women, establishing clear and delimited spaces for their involvement.

Mothers also posited how perception of competitiveness, the shyness, and breach of privacy through the presence of a male affected their engagement during the intervention, especially during group calls. These results are aligned with other evaluations of mHealth interventions in low-and-middle-income countries which confirm how fundamental perceived confidentiality and privacy are for user participation, influencing the acquisition of targeted behaviors [30]. One interviewee proposed ice-breaker games to increase interpersonal interactions. Another idea to promote increased participation from previous community-based interventions may be to use a participatory action-learning cycle strategy. In these, women in the groups could identify their current challenges as mothers and formulate strategies to overcome them, guided by the group intermediator [16, 24]. This participatory learning and action groups have improved maternal and neonatal health outcomes in a variety of settings, mainly through the enhancement of healthy behaviors [56].

Finally, one unexpected finding from this study was that the participants suggested various topics to add to the educational component. However, most of these topics were theoretically included in the planned list of topics the intervention aimed to deliver. This issue underlines the importance of analyzing the fidelity of the intervention, exploring what is being provided, and if the content given is following the program objectives [31]. This could be done by observation and checklists, as is recommended within implementation science approaches [31]. For example, it is possible that moderator discomfort with sensitive topics led them to be under emphasized or skipped ultimately.

This study contributes to the evidence on implementation factors needed to promote, sustain, and scale virtual maternal and newborn interventions like MeSSSSage, considering the contextual factors essential for success. Through a qualitative analysis of acceptability and perceived impact, it raises the voice of the participants, empowering women as active actors in the intervention, giving them the proper space for evaluation of the ongoing program. Moreover, evaluating what mHealth interventions work, how, and in which contexts is of great relevance today with the increasing use of technology as a resource to deliver health services after the COVID pandemic, providing fundamental information for scaling up, particularly in settings like rural India, with low access to health professionals [57, 58].

### Limitations

Interpretation of the present research findings should acknowledge certain limitations. First, given that the interviewers were affiliated with the institution that delivered the intervention, the participants might have been compelled to give positive feedback on MeSSSSage and underreport the negative aspects, a phenomenon known as social desirability bias [59]. Nevertheless, the fact that the interviewers were known to the women could have helped build rapport and facilitate conversation about personal experiences. In addition, during the selection process, there was concern about a possible “healthy user effect”, by interviewing only participants willing to answer the phone and who had favorable participation and opinion of the intervention, including its impact [60]. However, the level of activity and engagement within the different modalities were known for each participant and integrated into our purposive sampling strategy.

### Further research

After performing this qualitative analysis, the present study opens some interesting inquiries. Exploring the effectiveness of the intervention in terms of impact on maternal and child health indicators will be interesting, in addition to the participants’ perspectives. Furthermore, this will help to recognize the role of individual health knowledge and behaviors within structural determinants like health care access and factors therein. Even with the accelerated advances in technology and its contributions to the provision of health services, the question remains whether mHealth will be able to overcome the barriers of access and opportunity for the most vulnerable populations or will contribute to increasing health inequities. For MeSSSSage, most participants had their own telephones and were able to use them, either by themselves or with the help of a third party. It will be interesting to evaluate the use of these new modalities of postpartum care in a population with lower literacy in technology. A solid prior evaluation of the context and an iterative implementation process considering the actors’ voices will be essential.

## Conclusions

This study provides insights into the high acceptability and perceived impact of a novel digital, group support, postnatal care program in a rural setting in India, including the users’ perceived barriers to engaging with the intervention and possible solutions and recommendations to overcome them.

These findings will enable refinement of the intervention, providing a more robust framing for its scalability and long-term sustainability. On a larger scale, conclusions from this research provide new insights and encouragement to global stakeholders that aspire to improve maternal and neonatal outcomes in developing countries through mHealth and community-based programs.

## Figures and Tables

**Table 1.** Sociodemographic data of the participants involved in the qualitative assessment of MeSSSSage intervention acceptability.

Characteristic	Values	

**Age (years) median (IQR)**		27 (5)
**Time married (years) median (range)**		2.5 (1–10)
**Obstetric history, n (%)**		
	First pregnancy	8 (50%)
	More than one pregnancy	8 (50%)
	Miscarriages	3 (19%)
	Livebirth	6 (38%)
**Educational attainment**, n (%)		
	Primary	0 (0%)
	Secondary	8 (50%)
	Tertiary	8 (50%)
**Household below poverty line, n (%)**		4 (25%)
**Intergenerational household, n (%)**		13 (81%)

**Table 2.** Summary of the barriers and facilitators to engaging in MeSSSSage using the Realist Evaluation framework.

Realist framework component	Barriers	Facilitators
		
Context		
	• Network connectivity • Household responsibilities, including childcare	• Family buy-in, particularly from the husband and mother-in-law
Intervention		
	• Technical difficulties with mobile application • Lack of guidance regarding interaction with other users	• Features of the mobile App and group chats: repeat mode for audio messages and videos
Actors		
	• User literacy • Level of comfort	• Intervention moderator’s guidance on technological issues • User motivation

## Data Availability

The datasets used and/or analysed during the current study are available from the corresponding author on reasonable request.

## Abbreviations

IVR: Interactive Voice Recognition system
